# Indirect Enhancement of ALD Thin-Film Properties Induced by the ECAP Modification of an As-Extruded Mg-Ca Alloy

**DOI:** 10.3390/mi15081006

**Published:** 2024-08-03

**Authors:** Pi-Chen Lin, Jun-Yu Li, Hou-Jen Chen, Kaifan Lin, Miin-Jang Chen, Kun-Ming Lin, Hsin-Chih Lin

**Affiliations:** 1 Department of Materials Science and Engineering, National Taiwan University, Taipei 106319, Taiwan; royroy162000@gmail.com (P.-C.L.); ardor7201616@gmail.com (J.-Y.L.); f10527a06@ntu.edu.tw (H.-J.C.); kafainlin@gmail.com (K.L.); mjchen@ntu.edu.tw (M.-J.C.); 2 Department of Materials Science and Engineering, Feng Chia University, Taichung 407301, Taiwan; kmlin@fcu.edu.tw

**Keywords:** atomic layer deposition, equal-channel angular pressing, indirect film improvement, corrosion protection, magnesium–calcium alloy

## Abstract

The purpose of this study is to investigate the indirect effects on the properties of ZrO_2_ films deposited by atomic layer deposition (ALD) when an Mg-Ca alloy is modified through equal-channel angular pressing (ECAP) following extrusion. The study aims to understand how the increase in CaO content in the native oxide layer of the Mg-Ca alloy influences the crystallinity and defect density of the ZrO_2_ film. Consequently, the corrosion protection performance of the ZrO_2_ film is enhanced by 1.2 to 1.5 times. A reduction in the anti-scratch property of the ZrO_2_ film was also observed, with a critical load reduction of 34 μN. This research provides a detailed analysis of the modifications induced by ECAP on the as-extruded Mg-Ca alloy and its subsequent impact on the properties of the ZrO_2_ film.

## 1. Introduction

Magnesium alloys possess physical and mechanical properties akin to human bones, including comparable density and Young’s modulus [1]. Furthermore, as an essential element for the human body, magnesium can gradually degrade and be absorbed, rendering magnesium alloys highly suitable for use as human implants [2]. Biodegradable magnesium alloy implants have garnered significant attention as viable alternatives to stainless-steel and titanium alloy implants, as they obviate the need for subsequent implant removal surgeries. However, the chemical reactivity of magnesium alloys within the human body gives rise to an accelerated degradation rate, leading to inadequate support during the bone healing process [3]. Moreover, the rapid accumulation of hydrogen, a by-product of degradation, can engender adverse effects, such as tissue inflammation, when an excessive number of hydrogen bubbles forms [4,5]. Therefore, controlling the degradation rate of magnesium alloy implants within the human body to ensure optimal outcomes is seen as a crucial issue.

To mitigate the degradation rate of magnesium alloys, several potential solutions exist in the form of alloying element additions, implementation of deformation processes, and utilization of surface modification techniques. These approaches have been studied to enhance the corrosion resistance of magnesium alloys [6,7,8]. Among these methods, equal-channel angular pressing (ECAP) and atomic layer deposition (ALD) are widely used and hold significant importance.

ECAP is an effective plastic deformation process that can significantly refine the grain structure of magnesium alloys. The refined grain structure improves the corrosion resistance and provides a uniform microstructure, which is beneficial for subsequent surface modification techniques. For instance, the ultrafine-grained microstructure of the ZK60 alloy achieved via equal-channel angular pressing (ECAP) demonstrates improved corrosion resistance when compared to both the as-extruded and as-cast ZK60 alloy [9,10]. During the ECAP process, as the material is extruded through a corner, shear forces induce substantial plastic deformation while preserving a consistent cross-sectional area. This phenomenon leads to the refinement of the microstructure and the uniform dispersion of second-phase particles [11,12,13].

On the other hand, atomic layer deposition (ALD) has emerged as a promising technique for surface modification, which refers to an extrinsic way. ALD enables the deposition of a precise and uniform protective layer on magnesium metal surfaces, effectively mitigating corrosion and enhancing durability. This study focuses on using ZrO_2_ films due to their excellent corrosion resistance and biocompatibility. The advantage of ZrO_2_ films is their ability to form a uniform and dense protective layer on the magnesium alloy’s surface, significantly reducing the corrosion rate. Additionally, ZrO_2_ films exhibit good stability in biodegradable environments, reducing the generation of by-products during degradation and thereby minimizing the risk of tissue inflammation [14].

Several studies have demonstrated the efficacy of ALD in reducing corrosion rates and improving the corrosion resistance of magnesium alloys [15,16,17]. By tailoring the film composition and thickness at the atomic scale, ALD provides a viable approach to expanding the applications of magnesium in diverse industries.

In this study, we employed the ECAP and ALD techniques for Mg alloys to directly enhance the as-extruded magnesium–calcium (Mg-Ca) alloy, aiming to improve its mechanical properties and corrosion resistance. Most importantly, we observed that the microstructural modifications of the Mg-Ca alloy induced by ECAP indirectly led to further improvements in the characteristics of the ZrO_2_ films prepared by ALD on the alloy. Based on this, we investigated the effects of ECAP on the mechanical properties and corrosion resistance of ALD films and discussed the reasons behind the improved properties of the films through non-intrinsic processing methods.

## 2. Experimental Methods

### 2.1. Sample Preparation

The initial as-cast Mg-Ca alloy ingot, which consisted of 98.8 wt% Mg and 1.2 wt% Ca, was extruded at a temperature of 300 °C. The extruded material was subsequently sectioned into rods with a diameter of 25.4 mm. A portion of these as-extruded rods was subjected to the ECAP process, the secondary processing, through the B_C_ route with a channel angle of 120°, with 8 passes performed at a rate of 10.0 mm/s. The ECAP process was carried out for 4 passes at 250 °C initially, followed by another 4 passes at 230 °C. After ECAP treatment, both the as-extruded and ECAPed rods were cut into pieces, each with a thickness of 2.0 mm. The terms as-extruded and ECAPed Mg-Ca are denoted as “EXT Mg-Ca” and “ECAP Mg-Ca”, respectively, in this work. Before film deposition, the rough surface and native oxide layer of the as-extruded and ECAPed Mg-Ca alloy were removed by polishing the surface with SiC abrasive papers ranging from P240 to P4000. Subsequently, the samples were cleaned with acetone in an ultrasonic generator to remove organic matter and then dried with a nitrogen gun.

### 2.2. Atomic Layer Deposition

Plasma-enhanced atomic layer deposition (PE-ALD) was employed to deposit the ZrO_2_ film using the ALD system (Fiji, Ultratech, Santa Clara, CA, USA). The precursor utilized in the process was Tetrakis(dimethylamino)zirconium (TDMAZr, 99% Strem Chemicals, Newburyport, MA, USA), while oxygen plasma served as the reactant. The ALD system was maintained at a pressure of 3.1 × 10^−1^ Torr and a working temperature of 200 °C. In the first half-cycle of each ALD cycle, a 20 mTorr dose of the TDMAZr pulse was introduced, followed by a 15 s purging step with argon carrier gas. In the second half-cycle, oxygen plasma was exposed to the sample for 20 s, and a 20 s purging step with argon gas was subsequently performed. After depositing, the specimens were then purged with nitrogen and stored in a vacuum environment to ensure a clean and consistent surface before each test.

### 2.3. Characterization and Analysis

The effect of ECAP on the microstructures of the Mg-Ca alloys was investigated using the composition analysis and backscattered electron images were obtained with an electron probe microanalyzer (JEOL JXA-8530F Plus, JEOL Ltd., Tokyo, Japan). On the other hand, electron backscatter diffraction (JEOL JSM-7800F Prime, JEOL Ltd., Tokyo, Japan) was employed to analyze the grain size and stress distribution of the Mg-Ca alloys. For the analysis of the ZrO_2_ film on as-extruded and ECAPed Mg-Ca alloys, X-ray diffraction was conducted to measure crystal structure and crystallinity by using the XRD (RIGAKU TTRAX-3, Rigaku Corporation, Tokyo, Japan) analyzer with Cu Kα radiation (λ = 1.5406 Å). The diffraction was carried out in the grazing incidence mode with a fixed incident angle of 0.5°. The diffraction pattern was scanned from 20° to 80° with a recording interval of 0.04° at a scanning rate of 4°/min. The composition and defect amount of the ZrO_2_ film were analyzed by the results of electron configuration obtained from X-ray photoelectron spectroscopy (Thermo K Alpha, Thermo Fisher Scientific, Waltham, MA, USA). The film thickness was measured using the field-emission transmission electron microscopy (TEM) technique (FEI Tecnai G2 F20, Thermo Fisher Scientific, Hillsboro, OR, USA) after the TEM samples were prepared by a focused ion beam (FEI Helios 600i, Thermo Fisher Scientific, Hillsboro, OR, USA).

To evaluate the wear resistance of the atomic layer-deposited films, a Hysitron TI 980 TriboIndenter (Hysitron TI 980 TriboIndenter, Bruker Corporation, Minneapolis, MN, USA) with a conical diamond indenter (model TI-0040) was used. A progressive load ranging from 0 to 500 μN was applied to create a scratch of a 10 μm length. Subsequent scanning tunneling microscopy provided 3D surface scans, and scratch depth variations were analyzed using Gwyddion 2.66 software (Czech Metrology Institute, Brno, Czech Republic).

### 2.4. Corrosion Evaluation

The corrosion behavior of the samples was evaluated at 37 °C in a simulated body fluid solution (SBF) through both electrochemical and immersion tests. The composition of the SBF solution is referred to in reference [18]. In the electrochemical experiments, a three-electrode cell setup was employed. The sample served as the working electrode, the saturated calomel electrode (SCE) acted as the reference electrode, and a platinum sheet was used as the counter electrode (CE). Electrochemical impedance spectroscopy (EIS) and potentiodynamic polarization curves (PDP) were measured by using a potentiostat machine (VersaSTAT 3F, Ametek, Inc., Berwyn, PA, USA). The open circuit potential (OCP) of the system was recorded until it stabilized before each measurement, and the stabilization time for the OCP was approximately 60 min. For each set of experiments, three samples were used to determine the average parameters to ensure the reliability and reproducibility of the results.

During the EIS measurement, a sine wave AC voltage with an amplitude of 14 mV compared to the open circuit potential was applied to the sample. The impedance and phase angle responses were collected within the frequency range of 10^5^ Hz to 10^−2^ Hz under the AC voltage. The measured spectrum was fitted with a given equivalent circuit using ZSimpWin 3.30d software (Ametek Scientific Instruments, Oak Ridge, TN, USA). As for the PDP tests, current values were recorded at a scanning rate of 0.5 mV/s within the range of −0.4 V to 1.2 V vs. OCP (open circuit potential). The corrosion current density was calculated by Tafel equation using Nova 2.1 software (Metrohm Autolab B.V., Utrecht, The Netherlands), and the voltage range set for the Tafel interpolation calculation was ±120 mV. On the other hand, immersion tests, where samples were immersed in an SBF solution at 37 °C for 1 h, were utilized to evaluate surface change after corrosion.

## 3. Results and Discussion

### 3.1. Equal-Channel Angular Pressing on As-Extrude Mg-Ca Alloy

**Figure 1:**
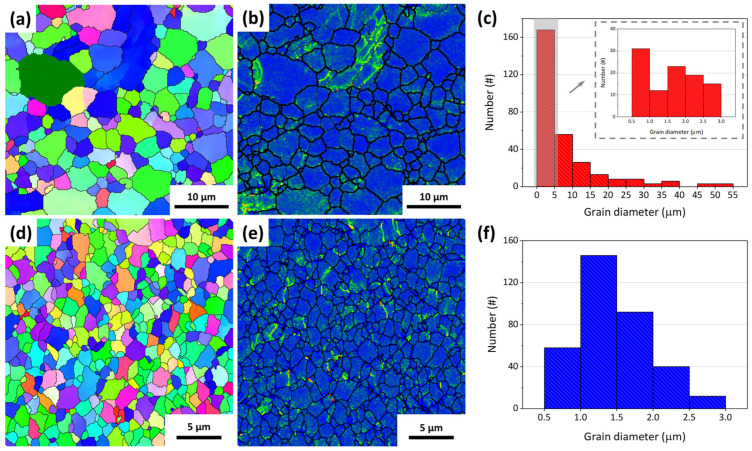
(**a**) Grain size distribution, (**b**) stress distribution, and (**c**) quantified number of grain sizes of the EXT Mg-Ca alloy. The inset (**c**) is the diameter distribution of grain that is smaller than 5 μm. (**d**) Grain size distribution, (**e**) stress distribution, and (**f**) quantified number of grain sizes of the ECAP Mg-Ca alloy measured from the EBSD.

**Figure 2:**
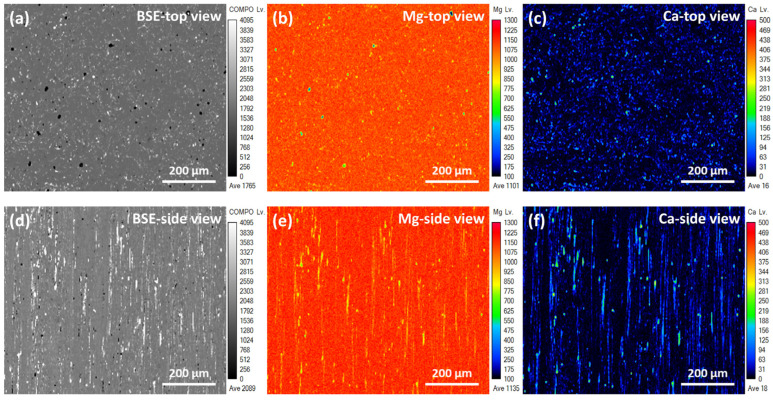
Images and element distribution of EXT Mg-Ca alloy. (**a**) Backscattered electron image, (**b**) element mapping of Mg, and (**c**) element mapping of Ca measured from the top-view direction. (**d**) Backscattered electron image, (**e**) element mapping of Mg, and (**f**) element mapping of Ca measured from the side-view direction.

**Figure 3:**
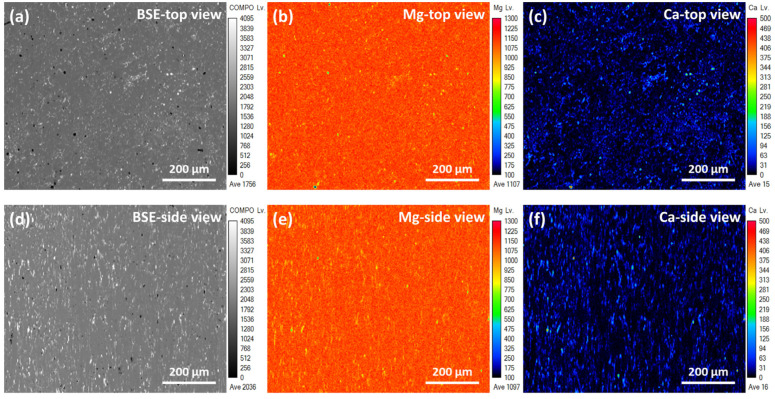
Images and element distribution of ECAP Mg-Ca alloy. (**a**) Backscattered electron image, (**b**) element mapping of Mg, and (**c**) element mapping of Ca measured from the top-view direction. (**d**) Backscattered electron image, (**e**) element mapping of Mg, and (**f**) element mapping of Ca measured from the side-view direction.

Plastic distortion is function to reduce the grain size of metal [19]. Figure 1 shows the morphology, remaining stress, and grain size distribution obtained for the EXT Mg-Ca and ECAP Mg-Ca alloys. As shown in Figure 1a,d, it can be seen that the EXT Mg-Ca alloy has a larger grain size than the ECAP Mg-Ca alloy. Additionally, stress concentrates within specific grains in the EXT Mg-Ca alloy, shown in Figure 1b, whereas stress is more evenly distributed across the ECAP Mg-Ca alloy, as seen in Figure 1e. The results of the qualified grain size distribution are shown in Figure 1c,f. The average grain size of the EXT Mg-Ca alloy measured from the EBSD is 5.02 μm, most of the grain sizes of the EXT Mg-Ca are smaller than 5 μm, and the diameters of some grains are in the range of 20 to 55 μm. After the ECAP process, the grain size of the Mg-Ca alloy is reduced to 1.48 μm, and just the grain that is smaller than 3 μm is observed. The 71% reduction in grain size of the ECAP Mg-Ca alloy occurs because the material undergoes severe plastic deformation at the intersection of the die corners during the equal-channel angular pressing process, leading to the gradual formation of dislocation walls due to the movement of numerous dislocations [20]. This results in the creation of grain boundaries, effectively dividing large grains into smaller ones achieving grain refinement. The residual stress distribution, as depicted in Figure 1b,e, shows a more uniform pattern of residual stress compared to the EXT Mg-Ca alloy. Figure 2 and Figure 3 display the backscattered electron images and elemental distribution from both the top view and side view of the EXT Mg-Ca and ECAP Mg-Ca alloys. In the top view, the direction is parallel to both the extrusion process and ECAP process directions. In the BSE images, as shown in Figure 2a,d and Figure 3a,d, the gray regions and white regions are observed for both the EXT Mg-Ca and ECAP Mg-Ca alloys. When comparing the BSE images with the results of the element distribution shown in Figure 2b,c,e,f and Figure 3b,c,e,f, the white regions in the BSE images show a higher Mg intensity than the white regions, and Ca intensity is only measured in the white regions, which indicates that the white regions observed represent the Mg_2_Ca intermetallic compound, which is the precipitate of the Mg-Ca alloy [21]. As shown in Figure 2 and Figure 3, the distribution of the Mg_2_Ca precipitate of the EXT Mg-Ca alloy presents a strip-shaped distribution. However, it mostly presents a finer distribution obtained from the ECAP Mg-Ca alloy, because the solid solution/re-precipitate’s behavior as a result of temperature increase and also the mechanical destruction caused by the channel corner underwent severe plastic deformation when the EXT Mg-Ca alloy passed through the ECAP channel [22]. In addition, through eight passes of the B_C_ route, the samples were subjected to severe plastic deformation in different directions, resulting in the uniform distribution of the Mg_2_Ca compound.

**Figure 4:**
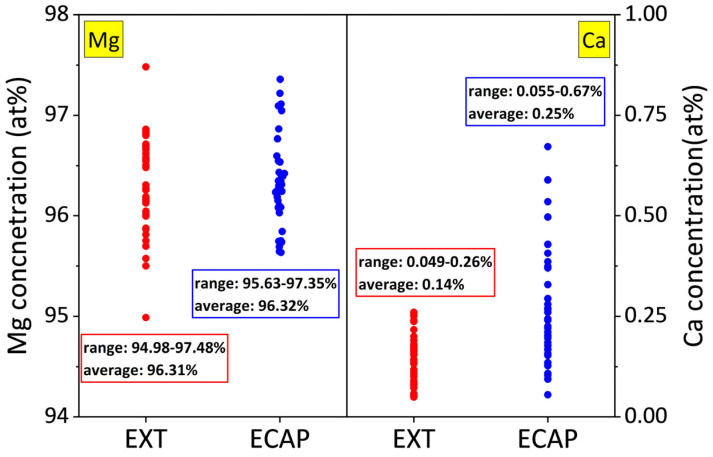
Concentrations of Mg and Ca measured from the α-Mg matrix region of EXT Mg-Ca and ECAP Mg-Ca alloys.

An Electron Probe Microanalyzer (EPMA) was used for the quantitative analysis of Mg and Ca concentrations in the α-Mg matrices of EXT Mg-Ca and ECAP Mg-Ca. The concentrations of Mg and Ca from 40 points are presented in Figure 4. The ranges of the concentration of Mg measured in both the EXT and ECAP Mg-Ca alloys are 94.98–97.48% and 95.63–97.35%, respectively, which are similar. Additionally, the average Mg concentration of the EXT and ECAP Mg-Ca alloys is around 96.32%. However, the range of the Ca element obtained from the EXT Mg-Ca alloy, being 0.049–0.26%, is narrower than the range of 0.055–0.67% that is the result of the ECAP Mg-Ca alloy. The average concentration of Ca in the α-Mg matrix of the EXT Mg-Ca alloy is determined to be 0.14 wt%, and the Ca concentration in the α-Mg matrix of the ECAP Mg-Ca alloy is 0.25 wt%. The results show that the Ca concentration of the Mg-Ca alloy increases after the ECAP process. The formation of the solid solution and re-precipitate occurs due to the temperature rise in the internal site of the sample, and the precipitate is destroyed by mechanical destruction, which leads to the re-dissolution of part of the Mg_2_Ca alloy into the α-Mg matrix so that an increase in Ca concentration is observed [23,24]. Additionally, defects and/or distorted structures induced by the ECAP process might have contributed to increasing the solid solubility of Ca atoms in the α-Mg matrix, leading to the formation of a supersaturated solid solution [25].

### 3.2. Characteristics of ZrO_2_ Film on the Mg-Ca Alloy

**Figure 5:**
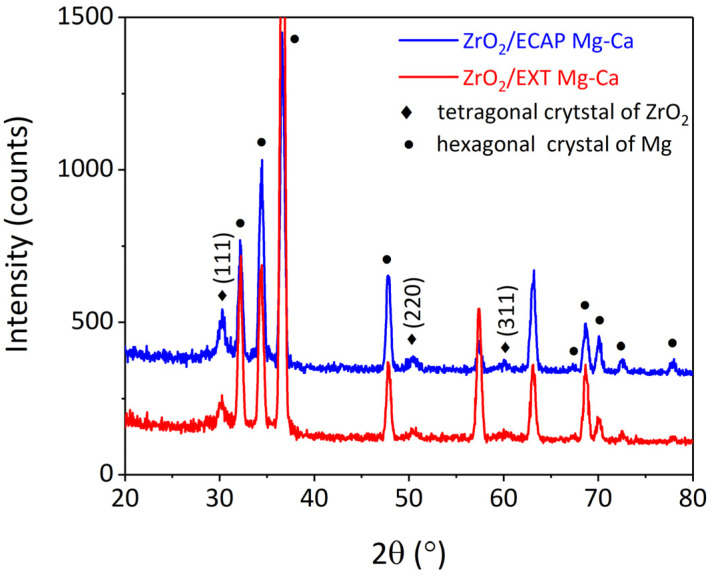
X-ray diffraction analysis of ZrO_2_/EXT Mg-Ca and ZrO_2_/ECAP Mg-Ca alloys.

The microstructure of ZrO_2_ films deposited on the Mg-Ca alloy by ALD was investigated using grazing-incidence diffraction-mode X-ray diffraction. As illustrated in Figure 5, the diffraction patterns of the ZrO_2_ films exhibit three diffraction peaks, which correspond to the (111), (220), and (311) planes of a tetragonal crystal structure [26], while other diffraction peaks arise from the Mg-Ca alloy. Notably, the peak intensities of the (111) and (220) planes in the ZrO_2_/ECAP Mg-Ca alloy sample are notably higher compared to those in the ZrO_2_/EXT Mg-Ca alloy. To facilitate the analysis of the crystalline characteristics of the ZrO_2_ films, quantitative data regarding the peak intensities of these diffraction peaks are integrated and provided in Table 1. Upon subjecting the Mg-Ca alloy to ECAP, the peak intensity of the (111) plane is increased from 168.7 to 210.1, and a slight increment is also measured from the intensity change in the (220) plane, which signifies that ZrO_2_ films deposited on the ECAP Mg-Ca alloy exhibit higher crystallinity in comparison to those on the EXT Mg-Ca alloy.

**Figure 6:**
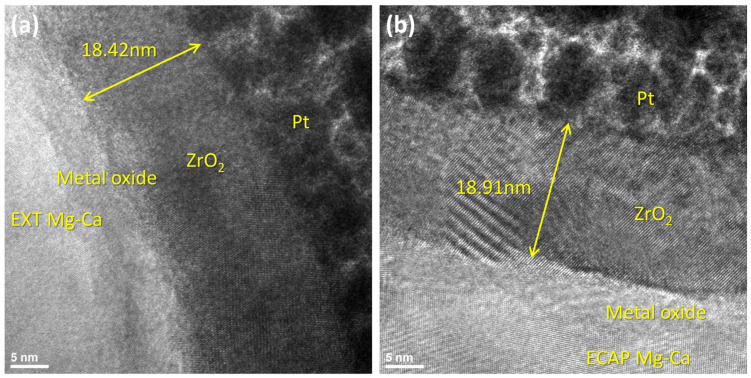
High-resolution TEM images of (**a**) ALD-ZrO_2_/EXT Mg-Ca and (**b**) ALD-ZrO_2_/ECAP Mg-Ca alloys.

The configuration and thickness of the ZrO_2_ film on the Mg-Ca alloy were assessed using TEM analysis. As illustrated in Figure 6, a metal oxide layer between the ZrO_2_ film and Mg-Ca alloy is observed, and the thickness of ZrO_2_ films on the EXT Mg-Ca and ECAP Mg-Ca alloy are 18.42 nm and 18.91 nm, respectively. This similarity in thickness indicates a consistent growth rate for ZrO_2_ films deposited on both EXT Mg-Ca and ECAP Mg-Ca substrates.

**Figure 7:**
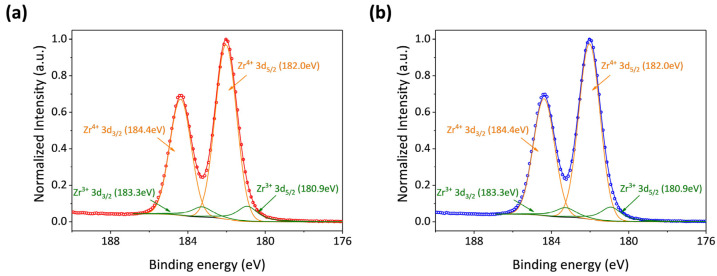
XPS results of Zr 3d XPS spectra of (**a**) ZrO_2_/EXT Mg-Ca and (**b**) ZrO_2_/ECAP Mg-Ca alloys. The experimental and fitting results correspond to the line with dots and bare lines.

In cases where the functional group reaction of TDMAZr is not entirely consummated during the ALD process, the formation of oxygen vacancies occurs [27]. These vacancies, in turn, induce modifications in the binding state of Zr. Once the Zr atoms have a neighboring oxygen vacancy, the number of valence electrons of Zr becomes 3 from 4 originally. Consequently, the relative proportions of Zr^4+^ and Zr^3+^ can offer insights into variations in the concentration of oxygen vacancies, serving as a proxy for the defects in the ZrO_2_ film [28]. Different binding energies manifest due to the distinct valence states and electronic configurations of Zr. Specifically, the electron binding energies for Zr^4+^ 3d_5/2_, Zr^4+^ 3d_3/2_, Zr^3+^ 3d_5/2_, and Zr^3+^ 3d_3/2_ are 182.0 eV, 184.4 eV, 180.9 eV, and 183.3 eV, respectively [29]. The Zr 3d X-ray Photoelectron Spectroscopy (XPS) spectra of ZrO_2_/EXT Mg-Ca and ZrO_2_/ECAP Mg-Ca alloys were analyzed and presented in Figure 7. The Zr^3+^ ratios extracted from the ZrO_2_ films on EXT Mg-Ca and ECAP Mg-Ca alloys amount to 10.80% and 10.09%, respectively, which means that ZrO_2_ film growth on the ECAP Mg-Ca alloy has less oxygen vacancy than on the EXT Mg-Ca alloy.

**Figure 8:**
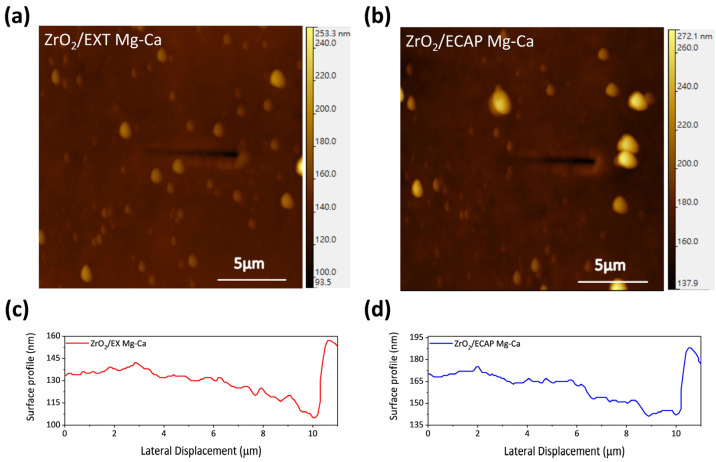
AFM image ofs (**a**) ZrO_2_/EXT Mg-Ca and (**b**) ZrO_2_/ECAP Mg-Ca alloys after scratching with nano-indenter. The depth profiles of the scratches of (**c**) ZrO_2_/EXT Mg-Ca and (**d**) ZrO_2_/ECAP Mg-Ca alloys.

**Figure 9:**
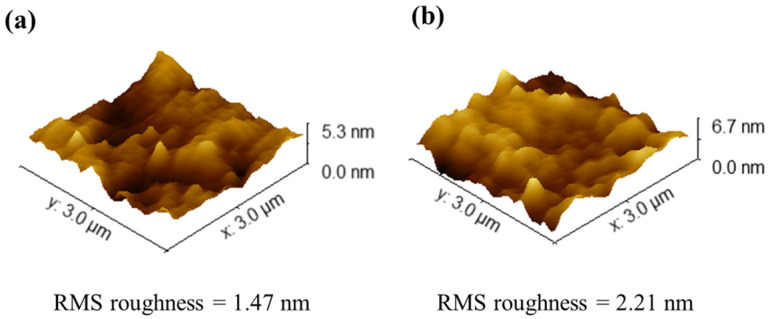
Surface images and the RMS roughness values of (**a**) ZrO_2_/EXT Mg-Ca and (**b**) ZrO_2_/ECAP alloys.

The nano-scratch test was employed to assess the wear resistance of the ZrO_2_ film. A 10 μm scratch was created on the surfaces of the ZrO_2_/EXT Mg-Ca and ZrO_2_/ECAP Mg-Ca alloys using a diamond indenter. The results of the nano-scratch tests are shown in Figure 8. For ZrO_2_/EXT Mg-Ca and ZrO_2_/ECAP Mg-Ca alloys, the depths of the diamond indenter’s penetration are divided into three stages: the first stage was approximately from 0 to 3 μm along the scratch, with an applied load of less than 100 μN. Here, depth changes were not pronounced, with surface height variations primarily arising from the surface morphology. In the second stage (around 3 to 6 μm), the diamond indenter penetrated into the film, with a depth maintained at around 5 nm. When the applied load reached a critical load, the film ruptured, and the diamond indenter started to penetrate into the Mg-Ca alloy substrate. Thus, the third stage (around 6 to 10 μm) shows a continuous increase in depth. The abrupt depth change at the boundary between the second and third stages is considered as the film rupture point, and the corresponding load was taken as the critical load of the film. The critical load for the ZrO_2_/EXT Mg-Ca alloy is 343 μN, higher than the critical load of 310 μN for the ZrO_2_/ECAP Mg-Ca alloy. The critical load is influenced by surface roughness and, therefore, the results of the surface roughness are depicted in Figure 9. According to the microstructure analysis of the XRD results, ZrO_2_ films on the EXT Mg-Ca alloy and on the ECAP Mg-Ca alloy exhibit different degrees of crystallinity. In line with the study by D. M. Hausmann and R. G. Gordon [30], higher crystallinity in the atomic layer-deposited ZrO_2_ films leads to greater surface roughness. Therefore, the rougher surface of ZrO_2_ film deposited on the ECAP Mg-Ca alloy results in a larger contact area with the diamond indenter, making the film more prone to delamination due to increased crystallinity.

### 3.3. Corrosion Characteristics

#### 3.3.1. Corroded Surface Morphology

**Figure 10:**
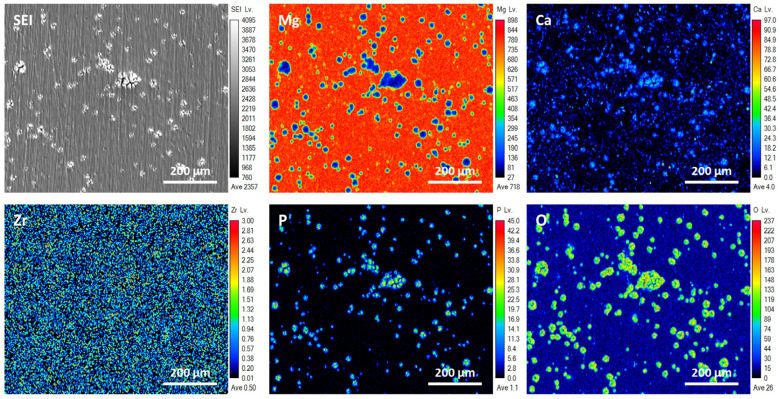
SEI images and elemental maps, including Mg, Ca, O, P, and Zr, of the ZrO_2_/EXT Mg-Ca alloy after immersion in SBF for 1 h.

**Figure 11:**
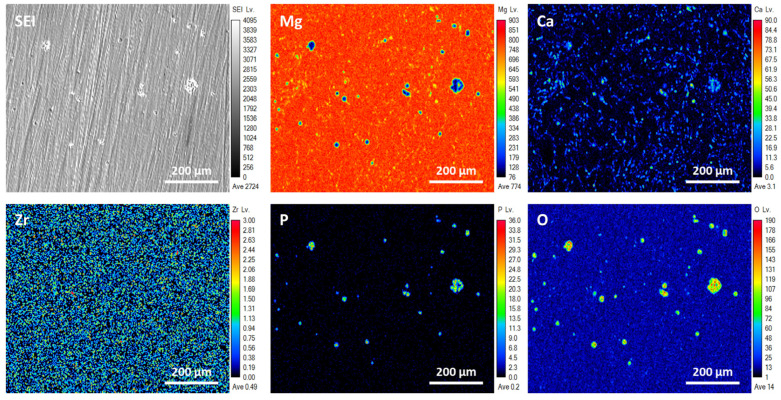
SEI images and elemental maps, including Mg, Ca, O, P, and Zr, of the ZrO_2_/ECAP Mg-Ca alloy after immersion in SBF for 1 h.

Figure 10 and Figure 11 depict secondary electron images and elemental distribution maps of ZrO_2_/EXT Mg-Ca and ZrO_2_/ECAP Mg-Ca alloy samples, respectively, subsequent to their immersion in simulated body fluid (SBF) at 37 °C. As shown in Figure 10 and Figure 11, distinctive volcanic cracks formed on the surface after the samples were immersed in SBF for 1 h, which is attributed to the chemical reaction that corrosive molecules, such as H_2_O and Cl-, react with for the Mg-Ca alloy. These agents engage in reactions with the alloy surface, leading to the gradual accumulation of by-products, notably hydrogen, at the interface between the ZrO_2_ film and the alloy [31]. The ZrO_2_ film is broken when it can no longer withstand the increasing hydrogen pressure, leading to the alloy being exposed to the surrounding solution. This exposure triggers a significant corrosion reaction, eventually causing the accumulation and subsequent rupture of corrosion products, leading to the formation of the observed volcanic cracks [32]. Additionally, the ZrO_2_/EXT Mg-Ca alloy’s surface exhibits a significantly greater volcanic crack density compared to the ZrO_2_/ECAP Mg-Ca alloy. Compared with the associated elemental mapping, a relatively low intensity of the Mg element is found in the volcanic crack regions. On the other hand, the areas where the ZrO_2_ film remains undamaged act as a protective shield, efficiently preventing corrosion and consequently preserving a higher intensity of the Mg element. Furthermore, elemental analyses identified the accumulation of diverse elements, including O, C, and P, in the volcanic crack regions. These elemental signals are ascribed to corrosion products like Mg(OH)_2_, CaCO_3_, and Ca_3_(PO_4_)_2_, which form due to the substrate’s oxidation reaction upon the interaction with SBF [33].

#### 3.3.2. Potentiodynamic Polarization

**Figure 12:**
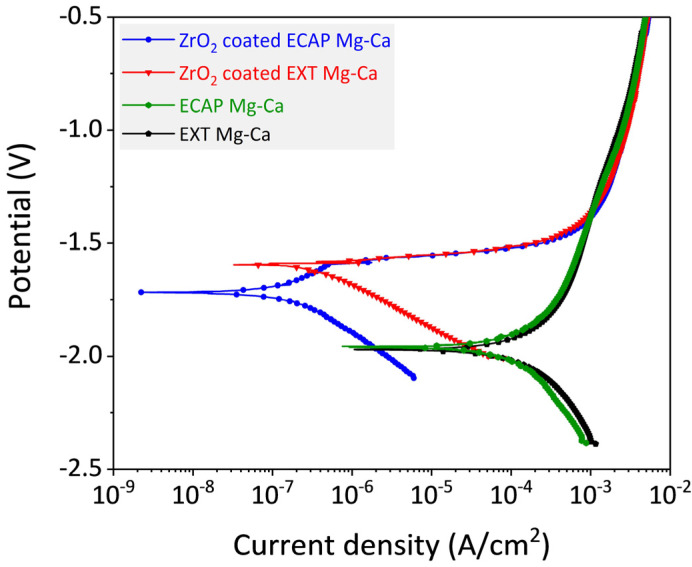
Potentiodynamic polarization curves of bare EXT Mg-Ca alloy and ECAP Mg-Ca alloy and ZrO_2_-coated Mg-Ca alloy.

The corrosion rate can be attained from the corrosion current density by measuring the potentiodynamic polarization (PDP) curves of ZrO_2_/EXT Mg-Ca and ZrO_2_/ECAP Mg-Ca alloy in SBF. Figure 12 shows the potentiodynamic polarization curves of bare EXT Mg-Ca and ECAP Mg-Ca alloys and a ZrO_2_-coated Mg-Ca alloy. The corrosion current densities (I_corr_) obtained by the Tafel extrapolation are listed in Table 2. The I_corr_ of the bare EXT Mg-Ca alloy is 9.39 × 10^−5^ A/cm^2^, and the I_corr_ of the bare ECAP Mg-Ca alloy decreases slightly to 7.66 × 10^−5^ A/cm^2^. However, under the protection of the ALD-ZrO_2_ film, the I_corr_ values of the EXT Mg-Ca and ECAP Mg-Ca alloys decrease to 4.67 × 10^−7^ A/cm^2^ and 1.34 × 10^−7^ A/cm^2^, respectively. It can be seen that the reduction effect of the ZrO_2_ film on the I_corr_ of the ECAP Mg-Ca alloy is 2.88-times higher than that of the EXT Mg-Ca alloy.

#### 3.3.3. Electrochemical Impedance Spectroscopy

**Figure 13:**
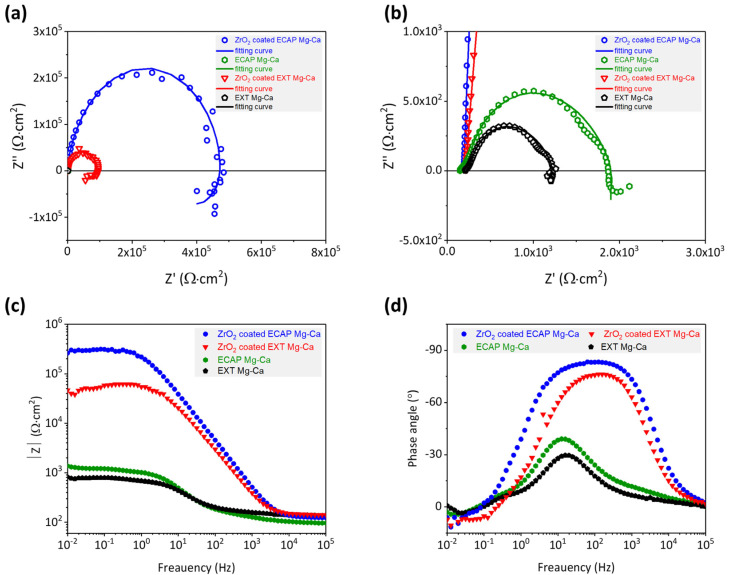
(**a**,**b**) Nyquist plots and Bode plots of (**c**) total impedance and (**d**) phase angle values of EXT Mg-Ca and ECAP Mg-Ca alloys with and without ZrO_2_ film deposition.

The reduction in corrosion current density indicates that the ALD-ZrO_2_ film enhances the corrosion resistance of the Mg-Ca alloy. Evaluating the corrosion protection effectiveness of the ALD-ZrO_2_ film involves electrochemical impedance spectroscopy. As shown in Figure 8, Nyquist plots and Bode plots illustrate the total impedance and phase angles obtained from the EXT Mg-Ca and ECAP Mg-Ca alloys with and without ZrO_2_ films. In Figure 13a, the Nyquist plots exhibit semicircular arcs, representing the capacitive behavior of the samples. Figure 13b is a local magnification of the high-frequency end of Figure 13a, highlighting the curves of uncoated samples. The extent of the loop in the curve corresponds to improved corrosion resistance [34]. Figure 13c exhibits the Bode plots indicating the variation in total impedance with frequency. The high-frequency region represents the impedance value of the corrosive solution. Since the same corrosive solution is used for measurements, the impedance values at a high frequency are very close. It is notable that the ZrO_2_/ECAP Mg-Ca alloy demonstrates a higher total impedance than the ZrO_2_/EXT Mg-Ca alloy in the low-frequency range, underscoring the superior corrosion protection offered by the ZrO_2_ film deposited on the ECAP Mg-Ca alloy [35,36]. By comparing the impedance values at a low frequency, the corrosion protection ability of the film can be inferred. Furthermore, the variation in phase angles also reflects the corrosion protection performance of the ZrO_2_ film. Figure 13d reveals that bare EXT Mg-Ca and bare ECAP Mg-Ca alloys exhibit phase angles of −29.7° and −39.0°, respectively. With the deposition of the ZrO_2_ film, the phase angle increased to −76.2° and −83.3°. Additionally, the phase angle of the sample was closer to −90°, which means more pronounced capacitance characteristics [37] and, hence, improved corrosion protection effectiveness. By fitting the curve in Figure 13a with the given equivalent circuit [38,39], a detailed analysis of the corrosion protection contribution of the ZrO_2_ film is conducted. As listed in Table 3, R_s_ signifies solution resistance, Q_f_ represents film capacitance, R_f_ denotes ZrO_2_ film resistance, C_int_ refers to the electric double-layer capacitance at the ZrO_2_ film and Mg-Ca alloy interface, R_t_ corresponds to the electron exchange resistance in the chemical reaction, L is the component that describes the species-absorbed interface inductance, and R_L_ is the resistance in series with L [29]. The smaller Q_f_ value for the ZrO_2_/ECAP Mg-Ca alloy and its higher n_f_ value suggest an improved capacitance quality, which aligns with the phase angle analysis results. The obtained improvement is attributed to the denser ZrO_2_ film on the ECAP Mg-Ca alloy because the enhanced crystallinity and lower oxygen vacancy concentration contribute to a denser film structure, which more effectively impedes the diffusion of corrosion ions toward the alloy [29,38]. Furthermore, to evaluate the corrosion resistance, polarization resistance (R_p_) is analyzed, and R_p_ accounts for both film resistance (R_f_) and charge transfer resistance (R_t_) [29,40]. The results are presented in Table 4. The ZrO_2_/ECAP Mg-Ca alloy demonstrates an increase in polarization resistance from 9.3 × 10^4^ Ω·cm^2^ to 4.8 × 10^5^ Ω·cm^2^, which signifies enhanced corrosion protection. Additionally, the heightened polarization resistance underscores the more effective corrosion protection provided by the ZrO_2_ film on the ECAP Mg-Ca alloy, resulting in the lower corrosion current density shown in Figure 12.

### 3.4. Discussion

**Figure 14:**
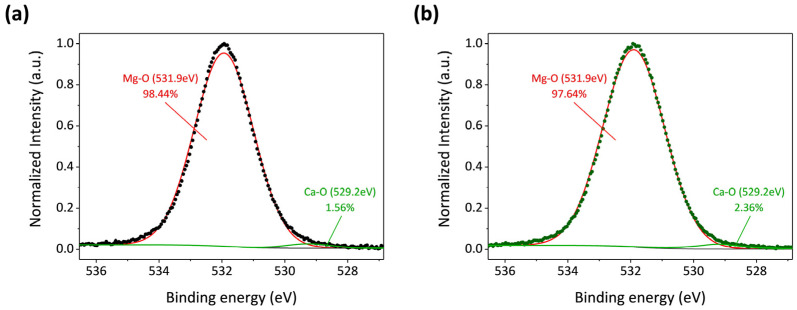
Fitting results of O 1s XPS spectra of native oxide layers of (**a**) bare EXT Mg-Ca and (**b**) ECAP Mg-Ca alloys.

From the above results, the ZrO_2_ films prepared by ALD are affected by different states of the Mg-Ca alloy, which is seen as an indirect influence. As shown in Figure 8, the anti-scratch property of the as-deposited ZrO_2_ film becomes worse once the Mg-Ca alloy is treated by ECAP processing, which shows that the decrease in the critical load from 343 μN to 310 μN was obtained from the nano-scratch test. However, the film quality of the ZrO_2_ film shows a great enhancement when the ZrO_2_ thin film is deposited on the ECAP Mg-Ca alloy. Figure 5, Figure 6 and Figure 7 show the increase in crystallinity and reduction in the oxygen defect, but the thickness remains similar. The density of tetragonal ZrO_2_ is 6.16 g/cm^2,^ and that of amorphous ZrO_2_ is 5.32 g/cm^2^ [41]. The crystallinity of the ZrO_2_ film increases, which means the film becomes denser [29,42]. In addition, the reduction in oxygen vacancies is accompanied by an increase in densification [43]. The results mean that film densification can be indirectly produced by processing the Mg-Ca alloy. On the other hand, the corrosion protraction property of the ZrO_2_ film is also observed, which is improved indirectly by the indirect effect. From the measurement results of EIS and PDP shown in Figure 12 and Figure 13, the ECAP process can directly enhance the corrosion resistance as well as the corrosion current density 1.2 times. However, the enhancement is increased by 1.5 times when the Mg-Ca alloy is covered by the ZrO_2_ film, which indicates the as-deposited ZrO_2_ film can provide additional corrosion protection. This result means the ZrO_2_ film on the ECAP Mg-Ca alloy has a better corrosion protection performance than the ZrO_2_ film deposited on the EXT Mg-Ca alloy. The densification of the ZrO_2_ film produced by the indirect effect from processing the Mg-Ca alloy leads to the inhibition of the penetration of corrosive ions, resulting in the additional corrosion protection provided by the ZrO_2_ thin film. The enhancement in corrosion protection is a result of the quality improvement induced by the indirect influence of the ECAP process on the Mg-Ca alloy. From the quantitative analysis of EPMA, as shown in Figure 4, the concentration of Ca measured from the α-Mg matrix of the EXT Mg-Ca alloy increases after the ECAP process, which leads to more Ca re-solute into the α-Mg matrix during the ECAP process and further results in the higher content of CaO in the native oxide layer than the bare EXT Mg-Ca alloy [44]. The native oxide layer of the Mg-Ca alloy is composed of MgO and CaO, which is naturally formed on the surface of α-Mg and intermetallic compound (Mg_2_Ca) [45]. The composition of the native oxide layer of the bare EXT Mg-Ca alloy and ECAP Mg-Ca alloy is measured by XPS analysis. As shown in Figure 14, the signal of the O bond obtained from both the bare EXT Mg-Ca alloy and ECAP Mg-Ca alloy is fitted with Mg-O and Ca-O bonds based on the above reference. The binding energies of Mg-O and Ca-O bonds have been studied at 531.9 eV and 529.2 eV, respectively [46,47]. To integrate the Mg-O and Ca-O peaks, the integrated peak intensity of CaO content in the native oxide layer of the ECAP Mg-Ca alloy is 2.36%, while that of the EXT Mg-Ca alloy is only 1.56%. Based on the thermodynamic calculation and experimental observation, Ca atoms tend to diffuse to the surface to form a CaO/MgO/Mg-Ca structure because of the large lattice distortion between the interface of CaO and α-Mg [44,48]. As presented in Figure 4, the α-Mg matrix of the Mg-Ca alloy has a higher Ca content so that more CaO is formed in the native oxide layer. In addition, the lattice constants of MgO and CaO are 4.18 Å and 4.86 Å, respectively, and the lattice constant of tetragonal ZrO_2_ is a = b = 5.09 Å [49,50], which means the lattice mismatch at the interface of ZrO_2_ and CaO should be less than ZrO_2_ and MgO. This result leads to the ZrO_2_ film being more likely to grow when it is deposited on the CaO layer [51]. Moreover, this more ordered stacking structure results in the effective adsorption of the precursors and reactants on the surface for a chemical reaction during the ALD process [52], which further improves film crystallinity and reduces the defective content of the film. Based on the results, ZrO_2_ film deposited on the ECAP Mg-Ca alloy shows a better corrosion protection feature than the ZrO_2_ film on the EXT Mg-Ca alloy due to the enhancement of film quality that is attributed to the reduced lattice mismatch between the ZrO_2_ film and native oxide layer with a higher CaO content.

## 4. Conclusions

On the one hand, an external ECAP process directly shrinks the grain size and reduces the remaining stress of the EXT Mg-Ca alloy. On the other hand, the properties of the ALD-ZrO_2_ film can be affected by processing the EXT Mg-Ca alloy with the ECAP technique. Through the ECAP process, the increase in the CaO content in the native oxide layer of the Mg-Ca alloy is caused by the resolution of Ca into the α-Mg matrix. The CaO layer is helpful for reducing lattice mismatch between the native oxide layer and ZrO_2_ film so that ZrO_2_ can grow on the Mg-Ca alloy with better film qualities, like increased crystallinity and less oxygen defects, which indicates the indirect enhancement is induced by the processing on the alloy. The film densification induced by enhanced crystallinity and reduced oxygen defect leads to the enhancement of corrosion protection provided for the Mg-Ca alloy from the ZrO_2_ film. However, the increase in the crystallinity is accompanied by a decrease in the anti-scratch property of the ZrO_2_ film. Overall, the ECAP processes on the EXT Mg-Ca alloy shows a negative impact on the anti-scratch property of the ZrO_2_ film, and it has the potential to be used to enhance film quality and corrosion protection properties.

## Figures and Tables

**Table 1:** The integrated intensities of (111), (220), and (311) XRD peaks of the ZrO_2_ film are shown in Figure 5.

Sample	(111)	(220)	(311)
ZrO_2_ film on EXT Mg-Ca alloy	168.7	73.0	77.3
ZrO_2_ film on ECAP Mg-Ca alloy	210.1	87.5	77.4

**Table 2:** Corrosion current densities of EXT Mg-Ca, ECAP Mg-Ca, ZrO_2_/EXT Mg-Ca, and ZrO_2_/ECAP Mg-Ca alloys.

Sample	EXT Mg-Ca	ZrO_2_/EXT Mg-Ca	ECAP Mg-Ca	ZrO_2_/ECAP Mg-Ca
I_corr_ (A/cm^2^)	9.39 × 10^−5^	4.67 × 10^−7^	7.66 × 10^−5^	1.34 × 10^−7^
E_corr_ (V)	−1.97	−1.61	−1.96	−1.72

**Table 3:** Fitting results of Nyquist plots of EXT Mg-Ca, ECAP Mg-Ca, ZrO_2_/EXT Mg-Ca, and ZrO_2_/ECAP Mg-Ca alloys.

Sample	R_s_ (Ω·cm^2^)	Q_f_ (F.s^−n^·cm^2^)	n_f_	R_f_ (Ω·cm^2^)	C_int_ (F.s^−n^·cm^2^)	R_t_ (Ω·cm^2^)	L (H·cm^2^)	R_L_ (Ω·cm^2^)
EXT Mg-Ca	208	8.4 × 10^−5^	0.60	1.5 × 10^2^	9.2 × 10^−6^	9.0 × 10^2^	5.1 × 10^4^	7.4 × 10^3^
ZrO_2_/EXT Mg-Ca	216	5.4 × 10^−7^	0.93	6.0 × 10^4^	6.5 × 10^−7^	3.3 × 10^4^	1.4 × 10^5^	3.0 × 10^3^
ECAP Mg-Ca	147	6.2 × 10^−5^	0.62	2.5 × 10^2^	9.2 × 10^−6^	1.6 × 10^3^	1.6 × 10^5^	3.0 × 10^−3^
ZrO_2_/ECAP Mg-Ca	190	2.9 × 10^−7^	0.96	3.3 × 10^5^	2.9 × 10^−7^	1.5 × 10^5^	2.3 × 10^6^	4.0 × 10^−2^

**Table 4:** Polarization resistance (R_p_) results of four samples. R_p_ is defined as the sum of film resistance (R_f_) and charge transfer resistance (R_t_), as shown in Table 3.

Sample	EXT Mg-Ca	ZrO_2_/EXT Mg-Ca	ECAP Mg-Ca	ZrO_2_/ECAP Mg-Ca
R_p_ (Ω·cm^2^)	1.1 × 10^3^	9.3 × 10^4^	1.9 × 10^3^	4.8 × 10^5^

## Data Availability

Data is contained within the article.
